# Mitochondrial J haplogroup is associated with lower blood pressure and anti-oxidant status: findings in octo/nonagenarians from the BELFAST Study

**DOI:** 10.1007/s11357-012-9444-4

**Published:** 2012-07-10

**Authors:** Irene Maeve Rea, Susan E. McNerlan, G. Pooler Archbold, Derek Middleton, Martin D. Curran, Ian S. Young, Owen A. Ross

**Affiliations:** 1 School of Medicine, Dentistry and Biomedical Science, Whitla Medical Building, Queen’s University Belfast, BT9 7BL Belfast, Northern Ireland UK; 2 Centre of Public Health, Queen’s University Belfast, Belfast, Northern Ireland UK; 3 Cytogenetics Laboratory, Belfast City Hospital, Belfast, Northern Ireland UK; 4 Biochemistry Laboratory, Belfast City Hospital, Belfast, Northern Ireland UK; 5 Transplantation Centre, Liverpool, UK; 6 Health Protection Agency, Addenbrookes Hospital, Cambridge, UK; 7 Mayo Clinic Jacksonville, Jacksonville, FL USA

**Keywords:** Blood pressure, J mitochondrial haplogroup, Longevity, Antioxidant status, Glutathione peroxidase activity, Vitamins A, E, C, α and β carotene, Urate

## Abstract

Mitochondria produce cellular energy but also free-radicals, which damage cells despite an array of endogenous anti-oxidants. In Northern Europe, the mitochondrial haplogroup J has been related to longevity in nonagenarians and centenarians but also with age-related disease. Hypertension is an important contributor to atherosclerotic-related diseases and its pathogenesis is associated with increased oxidative stress. In this study, we questioned whether J haplogroup octo/nonagenarians from the Belfast Elderly Longitudinal Free-living Elderly STudy (BELFAST) study showed evidence of protective blood pressure or anti-oxidant profile which might explain their longevity advantage. Briefly, in a cross-sectional study, community-living, mentally alert (Folstein >25/30), octo/nonagenarian subjects, recruited for good health, were enlisted and consented as part of the BELFAST study, for blood pressure, anthropometric measurements and blood sampling. DNA typing for mitochondrial haplotypes was carried out with measurements for enzymatic and non-enzymatic antioxidants. J haplogroup carriers showed lower systolic blood pressure and glutathione peroxidase activity (Gpx) with higher folate measurements. There was no change in urate, bilirubin, albumin or nutrition-related antioxidants-selenium or vitamins A, C and α and β carotene. BELFAST study mtDNA J haplogroup octo/nonagenarians showed lower blood pressure and reduced glutathione peroxidase activity and higher folate, but no change for other antioxidants. These findings are of interest in view of mtDNA J haplogroup’s association with increased age in some previous studies.

## Introduction

Mitochondria undertake multiple critical functions in a cell. They are the chief source of efficient energy production in cells, converting glucose into numerous high-energy phosphate bonds which transform adenosine diphosphate (ADP) to adenosine triphosphate (ATP) along the electron cascade. In addition to generating much of the cellular energy, mitochondria regulate the cellular redox state, produce most of the cellular reactive oxygen species (ROS), buffer cellular Ca2+ and initiate cellular apoptosis. Mitochondria have limited protection from oxidative stress (Wallace 1992) and energy production comes at the price of free radical production which risks damaging DNA, proteins, lipids and carbohydrates within the mitochondria, the cytoplasm, nucleus and cell membrane, with knock-on negative effects on cellular transcription and metabolic pathways (Gómez and Hagen 2012). As a counter-balancing mechanism, the cell has a range of enzymatic (glutathione peroxidase, catalase and superoxide dismutase), non-enzymatic (glutathione, albumin, bilirubin and uric acid) (Meister 1994) and nutrition-related antioxidant mechanisms (vitamins A, E and C together with selenium), (Padayatty et al. 2003; Dragsted et al. 2004) which together and separately, help minimise oxidative damage (Miquel 2002).

As we age, the free radical theory of ageing (Harman 1956) suggests that cells become less efficient at managing cellular damage, because the burden of damage becomes too great, there is inadequate antioxidant capacity, compromised nutrition or a combination of all three. The patho-physiology of a whole range of human age-related and degenerative diseases as well as cancer, have been held to be caused by an age-related decline in our ability to manage our free radical damage at the cellular level (Mueller et al. 2012; Mancuso et al. 2007; Chinnery et al. 2000; Cadenas and Davies 2000; Harrison et al. 2003).

In animal studies, nuclear and mitochondrial genes seem to both have an influence on the rate of ageing and free radical burden. In mice with genetically increased enzymatic anti-oxidant catalase activity, there is evidence for reduced atherosclerosis and increased lifespan (Cutler 2005) and mitochondrial-related genes affect oxidative stress in mice (Thompson 2006). Unlike nuclear genes which are protected by histones, mitochondria are extremely susceptible to oxidative damage. Between 2 and 4 % of the oxygen consumed by mitochondria is converted into superoxide anions by the electrode transport chain (Wallace 1992) with the consequence that mitochondrial mutations are relatively common. These mutation differences can define people and population clusters and their genetic heritage. Each person can be described by a mitochondrial haplogroup which is defined by a series of mutation changes which have been inherited by groups of people or populations, albeit several generations previously. Most Europeans are considered to derive from the main mitochondrial mitochondrial haplogroup root called *R* which has branching subgroups of U (U5, U6, U2, U, U8 and K), V and H, T and J, and B. The mitochondrial haplogroups J and T therefore share the same sub-branch of *R* and are considered to have been inherited from one woman, who is thought to have belonged to the hunter–gatherers that colonised Europe thousands of years before the agricultural revolution moved across Europe.

In extreme longevity, there is some suggestive evidence that mutations in the mitochondrial genome might play a role in *‘successful’* ageing. The Caucasian haplogroup J is characterised by the mutations T489C, A10398G, A1262G, G13708A plus the T4216C, A11251G and C15452A substitutions which are shared with haplogroup T. The J combination of mutations seem to confer a higher chance of achieving longevity than other mtDNA haplogroups in Northern Italians (De Benedictis et al. 1999), Northern Irish (Ross et al. 2001), Finns (Niemi et al. 2003) and Northern Spaniards (Domínguez-Garrido et al. 2009) but has have not been corroborated in Southern Italians (Dato et al. 2004), in a Tunisian population (Cherni et al. 2009) and in central Spaniards (Pinós et al. 2012), suggesting that the J haplogroup association may be population-specific. Other issues such as study differences in the ethnic and geographic origins of subjects and age differences of the ‘longevity’ cohorts are also potential confounders, since some reports studied centenarians (De Benedictis et al. 1999; Pinós et al. 2012), other studies younger elders, i.e. 85+ years (Domínguez-Garrido et al. 2009); 80–97 years (Ross et al. 2001) or 90–91 years (Niemi et al. 2003).

In seeking a possible explanation for these varied findings, Ruiz-Pesini et al. 2004, suggested that the clustering of J mitochondrial haplogroups in colder northern European climes might track with an evolutionary advantage relating to improved mitochondrial energy and therefore heat production, but at the price of increased anti-oxidant stress and the predilection for degenerative diseases in unfavourable cellular environments. Alongside the evidence relating the J mitochondrial haplogroup with longevity, J and related haplogroups have conversely also been variously associated with degenerative diseases including Parkinsons disease (Ross et al. 2003; Mancuso et al. 2007). Additionally, the mitochondrial haplogroups are implicated in clinical manifestations related to atherosclerosis or hypertension (Rosa et al. 2008; Rybka et al. 2011a). Both hypertension and atherosclerosis are increasingly considered to have a stress-related pathogenesis (Schnabel and Blankenberg 2007; Harrison et al. 2003) with broad evidence that measures anti-oxidant status, e.g. glutathione peroxidase activity (Gpx) may be increased in these situations or conversely reduced where cardiovascular disease and hypertension is minimal.

In this study, we tested the hypothesis that Belfast Elderly Longitudinal Free-living Elderly STudy (BELFAST) octo/nonagenarians carrying the mitochondrial J haplogroup would show a better blood pressure and antioxidant phenotype as a part explanation of their good-quality longevity. We also carried out comparisons of the T mitochondrial haplogroup which shares a similar mitochondrial subgroup root as J, though a number of additional mutations define a separate branch.

## Methods and materials

### Subject phenotype characteristics

#### Subjects

**Fig. 1:**
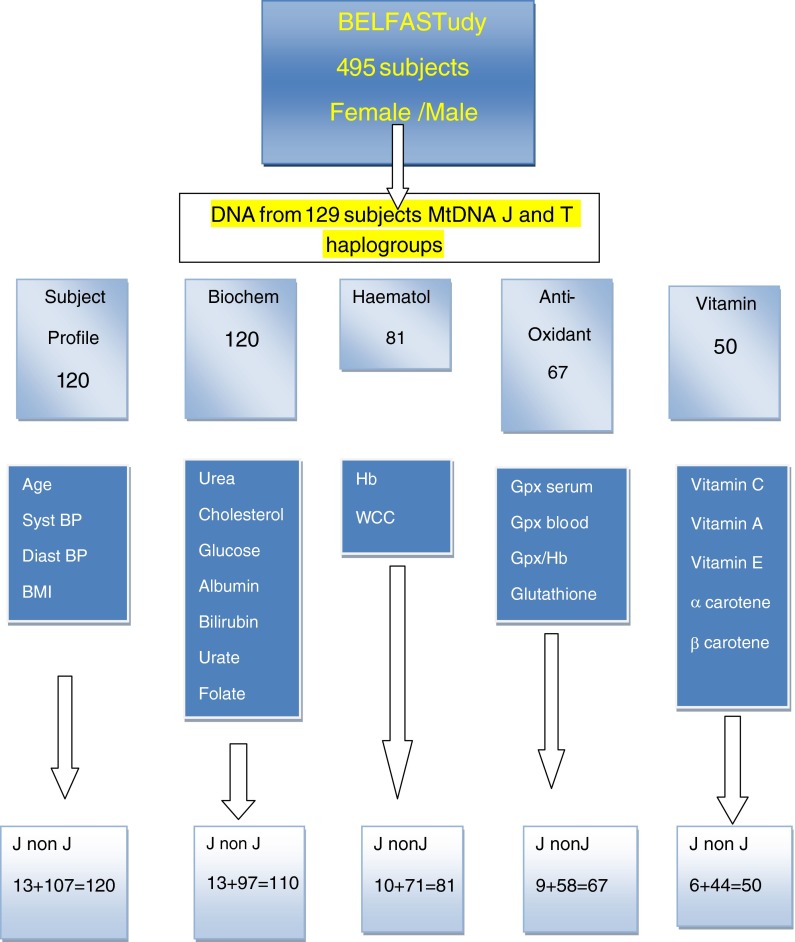
Flow diagram for subject base for BELFAST octo/nonagenarians with mtDNA, anthropometric, biochemical, haematological and antioxidant sample paths

Elderly subjects were a consecutive mid-study cohort enlisted from the BELFAST study, aged 80–100 years of age (Rea 2010; Rea et al. 2009), who met SENIEUR protocol (Ligthart et al. 1984). At recruitment, all subjects were mentally alert (Folstein >25/30), (Folstein and Folstein et al. 1975), apparently well and living in the community. One hundred and twenty-nine elderly subjects had DNA available for characterisation of J and non-J mitochondrial haplogroups (Ross et al. 2001). A flow diagram Fig. 1 shows the numbers of matching anthropometric variables, biochemistry, haematological, antioxidant and vitamin measurements which were available for the subjects. Because of subject frailty or inadequate samples, not all subjects have complete matching data. All subjects gave written consent and Ethical Consent for the study was obtained from Ethical Committee of Queens University Belfast.

#### Blood pressure measurements and sample collection

Subjects were seen in their own homes by the trained research officer and nurse. Blood pressure was measured in a sitting position and after 5 min of rest using a mercury sphygmomanometer, following the recommendations of the British Hypertension Society. Systolic blood pressure and diastolic blood pressure were measured using Korotkoff phase I and phase V, respectively. Blood was collected from subjects at a morning visit and returned to Belfast City Hospital Laboratories within 1 h of collection for various analyses.

### Methods

#### Glutathione (GSH)

GSH blood samples, freshly collected into metaphosphoric acid-containing tubes, were transported in liquid nitrogen and stored at −70 °C until analysis by High Performance liquid Chromatography.

#### Glutathione peroxidise

Gpx was measured in serum using a modification of the coupled assay of Paglia and Valentine as described by McMaster et al. 1990.

#### Selenium in serum and whole blood

Selenium in serum and whole blood was measured by atomic absorption spectrophotometer (McMaster et al. 1990). The intra-assay reproducibility for serum selenium showed a coefficient of variation (CV) for between-runs of 5.7 and 4.4 % within run. For selenium in whole blood the CV was 5.9 % between runs and 5.7 % within run.

#### Vitamins A, C and E and α and β carotene

Vitamins A, C and E and α and β carotene were measured in serum collected and stored at −70 °C and measured by standard methods (Ulker et al. 2004).

#### Uric acid and bilirubin

Uric acid and bilirubin were collected and routinely measured daily by Belfast City Hospital automated multichannel analyser.

### Genotype characteristics

#### DNA separation, mitochondrial haplotyping and phylogenetic analysis

DNA was separated from whole blood by standard salting out method (Miller et al. 1988). Briefly, PCR primers employed for amplification of the 2,643-bp region of interest in the mitochondrial genome were those described by Torroni et al. (1994) but extended at the 5′ end to improve the specificity and sensitivity of the PCR as noted previously (Ross et al. 2001; De Benedictis et al. 1999).

The phylogenetic relationships between the 46 haplogroups observed in this study were analysed using maximum parsimony criteria, using the cluster of programmes in the PHYLIP package (Version 3.5c; Felsenstein 1993) and as previously described (Ross et al. 2001).

### Statistical analysis

For each variable, values are expressed as mean and standard deviation (SD) or as medians for non-normal distributions. Subject age, biochemical variables, Gpx activity and the other antioxidants were categorised by J or non-J mitochondrial haplogroup status and analysed by *t* test or Mann–Whitney *U* (mwu) as appropriate. Blood pressure and a number of related variables were also categorised and comparison made for T and non-T mitochondrial haplogroups. Logistic regression analysis and odds ratio (OR) was used to predict differences in blood pressure categories defined by mitochondrial groups J non-J and T non-T and multiple models were constructed and analysed to include variables significantly different or close to significance in *t* test or mwu or other analyses. A *p* value of <0.05 was regarded as significant.

## Results

### Subject phenotype characteristics

#### Subjects

Subject characteristics are described in Tables 1, 2 and 3. Mean age was 89.6 [SD 4.6] years with no difference between J and non-J mitochondrial haplogroup carriers. Haemoglobin, white cell count, glucose, cholesterol and measures of renal function were not different between J and non-J haplogroup carriers. There was also no significant difference for body mass index and cholesterol.

**Table 1:** Phenotypic characteristics of subjects categorised by J and non-J mitochondrial haplogroup

	J haplogroup	Non-J haplogroup	*t* test
Age, years	88.1 (13) [4.2]	89.9 (113) [4.5]	0.12
Sex	5F/8M	80F/33M	
BMI, kg/m^2^	24.0 (12) [5.0]	23.8 (104) [3.7]	0.88
Glucose, μmol/l	5.6 (12) [1.2]	6.1 (97) [2.7]	0.54
Cholesterol	4.9 (12) [1.2]	5.5 (97) [1.1]	0.10
Urea, μmol/l	9.2 (12) [6.4]	7.3 (97) [2.6]	0.06
Haemoglobin, g/dl	13.0 (10) [1.2]	13.4 (71) [1.3]	0.29
White blood cell × 10^9^	8.1 (10) [3.9]	6.9 (71) [1.8]	0.10

Number of subjects are in round brackets, standard deviations are in square brackets

*BMI* body mass index

**Table 2:** Systolic and diastolic blood pressure, glutathione peroxidase activity, glutathione, and selenium categorised by J and non-J mitochondrial haplogroups

	J haplogroup	Non-J haplogroup	*t* test
Systolic blood pressure, mmHg	122 (13) [14]	136 (107) [18]	0.01*
Diastolic blood pressure, mmHg	76 (13) [14]	82 (107) [12]	0.08
Selenium μmol/l (serum)	0.75 (11) [0.23]	0.82 (89) [0.26]	0.41
Glutathione peroxidase activity (serum)	193 (7) [71]	259 (58) [55]	0.01*
	*186*	*256*	0.03*
Glutathione peroxidase activity (blood)	3,694 (7) [822]	4,373 (58) [741]	0.03*
	*3,333*	*4,311*	0.01* ω
Glutathione peroxidase activity/gmHg (blood)	32.3 (7) [3.3]	35.0 (58) [6.3]	0.31
Glutathione	9.9 (5) [2.5]	10.1 (30) [2.2]	0.89

Number of subjects are in round brackets, standard deviations are in square brackets

*Italic* median and ω mwu

*p < 0.05

**Table 3:** Serum antioxidants categorised by J and non-J mitochondrial haplotype

	J haplogroup	Non-J haplogroup	*t* test mwu ω
Albumin, g/l	39.9 (12) [5.0]	40.3 (94) [3.3]	0.72
Bilirubin, μmol/l	10.4 (12) [4.4]	10.5 (94) [4.3]	0.97
Folate, μg/l	7.9 (12) [5.4]	5.6 (94) [3.5]	0.05*
Urate, μmol/l	*0.24* (6) *[0.13–0.56]*	0.25 (44) *[0.12–0.55]*	*0.64 ω*
Vitamin A, μmol/l	*2.3 (6) [1.0–3.6]*	*2.0 (44) [0.8–7.0]*	*0.89 ω*
Vitamin E, μmol/l	*20.3 (6) [13–32]*	*26.3 (44) [10.6–57.6]*	0.05* ω
α Carotene, μmol/l	*0.11 (6) [0.01–0.36]*	*0.16 (44) [0.01–1.2]*	*0.27 ω*
β Carotene, μmol/l	*0.22 (6) [0.03–2.4]*	*0.42 (44) [0.04–1.8]*	*0.73 ω*
Vitamin C, μmol/l	*11.5 (6) [6–59]*	*32.9 (28) [1–87]*	*0.21 ω*

Number of subjects are in round brackets, standard deviations are in square brackets

*Italic* median, range and Mann–Whitney *U* ω

*p < 0.05

#### Medication and smoking

There were differences between the use of medications across J and non-J mtDNA haplogroups. It was more common for J mtDNA haplogroup carriers compared to non-J mtDNA haplogroups to be on no medication, overall 20 viz 10 %, respectively. Anti-hypertensive use was 32 vs 32 % respectively and aspirin use 0 vs 14 % was more common in non-J mtDNA haplogroup subjects compared to J mtDNA haplogroup subjects.

With respect to smoking there was little difference in the categories of never, past and current smokers between J and non-J mtDNA haplogroup carriers with 40 vs 50 % of subjects in J and non-J mtDNA groups advising that they had never smoked, 20 vs 21 % saying they had smoked in the past and 20 vs 21 % saying they were current smokers.

### Genotype characteristics

#### Mitochondrial J haplogroup and blood pressure

**Fig. 2:**
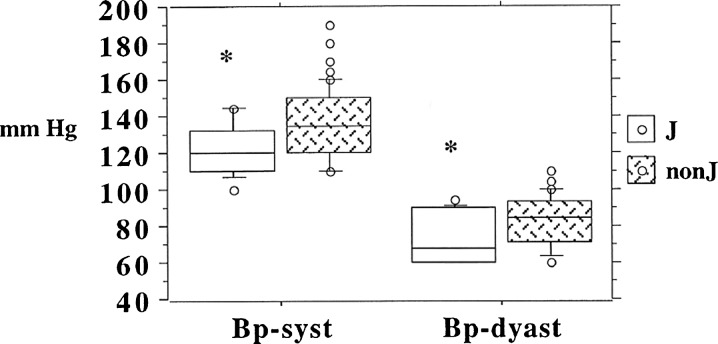
Systolic and diastolic blood pressure categorised by mtDNA J and non-J haplogroups for BELFAST octo/nonagenarians with *box* and *whisker plots* showing median and 25th and 75th percentiles

**Fig. 3:**
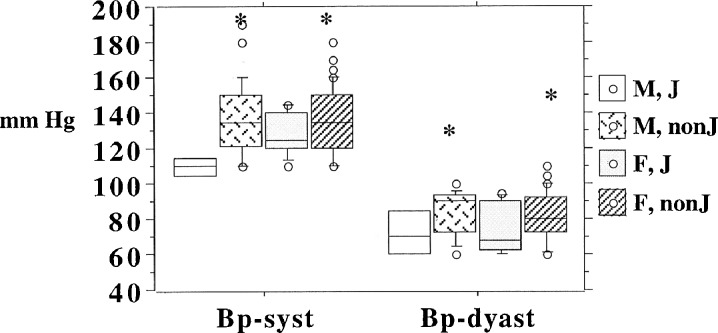
Systolic and diastolic blood pressure categorised by mtDNA J and non-J haplogroups for male and female BELFAST octo/nonagenarians with *box* and *whisker plots* showing median and 25th and 75th percentiles

Mean systolic blood pressure across the octo/nonagenarian group was 132 [standard deviation (SD) 17] mmHg, with a significantly lower value (*p* = 0.02) for J haplogroup 122 [SD 14] compared to non-J haplogroups 136 [SD 18]. Diastolic blood pressure was also lower in J haplogroup octo/nonagenarians, 76 [SD 14] mmHg, compared with 82 [SD 12] mmHg, in non-J haplogroups Table 2 and Fig. 2, but this difference just failed to reach significance (*p* = 0.06). A similar pattern was represented across male and female, J and non-J haplogroups for both systolic and diastolic blood pressure, Fig. 3.

#### Mitochondrial T haplogroup and blood pressure 

Systolic and diastolic blood pressure were not significantly different when categorised by mitochondrial haplogroups T non-T; systolic blood pressure 133 [SD 11] and 134 [SD 18] *p* = 0.99 for T and non-T, respectively, and for diastolic 84 [SD 5] and 81 [13] *p* = 0.80, respectively (data not shown).

#### Mitochondrial J haplogroup and cellular anti-oxidants 

Glutathione peroxidase activity in serum and blood categorised according to J and non J haplogroup carrier is shown in Table 2. Mean glutathione peroxidase activity in blood and serum was significantly lower in J carriers (*p* = 0.03 and *p* = 0.01, respectively). There was no change in serum glutathione or selenium between J and non-J carriers.

#### Non-enzymatic and nutrition-related antioxidants and mitochondrial J haplogroups

The non-enzymatic antioxidants albumin, bilirubin and urate were not significantly different when subjects were categorised according to mitochondrial J haplogroup, Table 3. Similarly there are no significant differences for serum vitamins A and C and α and β carotenoids, for J and non-J mitochondrial haplogroup carriers, though numbers are small. Serum folate was significantly higher in J compared to non J haplogroup carriers and Vit E lower in the small number of J haplotype carriers.

## Logistic regression

Logistic regression was used to further explore whether carriage of the J or the non-J mitochondrial haplotype by BELFAST octo/nonagenarians could predict blood pressure and associated anti-oxidant status since logistic regression does not depend on the normality of independent variables nor the linearity of relationships. Table 4 shows coefficients B, *χ* ^2^ and associated *p* values with Exp(B) as the odds ratio (OR), using J and non-J mitochondrial status as a dichotomous variable for prediction of blood pressure, Gpx and folate-variables previously noted to be significantly different between the J mitochondrial categories. For systolic blood pressure, J carriers were predicted to be likely to have lower blood pressure compared to non-J carriers by about 5 mms for each 100 mmHg (OR 0.95, confidence limits CI 0.91–0.99; *p* = 0.017) which appeared to the confirm findings from the preliminary comparative analyses. For the anti-oxidant glutathione peroxidise activity, J mitochondrial carriers were also predicted to have somewhat lower glutathione peroxidase activity (OR 0.98; CI 0.96–1.0; *p* = 0.027) in agreement with previous findings. Folate by contrast showed a trend for higher values in J haplotype carriers (OR = 1.12; CI 0.99–1.27; *p* = 0.07). Multinominal logistic regression models 1, 2 and 3 were set up with J and non-J mitochondrial haplotype as the dichotomous variable to include the independent variables blood pressure, glutathione peroxidase activity and folate. Table 4 shows the log likelihood ratio model fit for each with associated *χ* ^2^, *p* and *r* ^2^ values and shows that while all models could predict the class of J or non-J mitochondrial haplotypes for all three independent variables, model 3 performed best in predicting class category for both blood pressure and glutathione peroxidase activity (*p* = 0.02). There was a suggestion that there may have been collinearity between glutathione peroxidase activity and folate though the *r* value −0.284 in the correlation matrix was not particularly high (data not shown).

**Table 4:** Logistic regression for J and non-J mitochondrial haplogroups for Systolic Blood Pressure, Glutathione Peroxidase activity and Folate for BELFAST octo/nonagenarians with best fit Multinominal Models 1, 2 and 3

Variables	J/nonJ	Coeff B	SE	Chi-sqare	*p* value	Exp(B)	CI (95%)
Syst BP (120)	13/107	−0.05	0.02	5.69	0.017*	0.95	0.91–0.99
Gpx (65)	7/58	−0.02	0.01	4.87	0.027*	0.98	0.96–1.0
Folate (106)	12/94	0.12	0.06	3.31	0.07	1.12	0.99–1.27
							
Model 1	Syst BP, Gpx, Folate		LR whole model fit for J/nonJ	*x* ^2^	9.89	*p* = 0.02*	*r* ^2^ = 0.29 predicts 95%
Model 2	Syst BP, Folate		LR whole model fit for J/nonJ	*x* ^2^	9.92	*p* = 0.007*	*r* ^2^ = 0.10 predicts 87%
Model 3	Syst BP, Gpx		LR whole model fit for J/nonJ	*x* ^2^	7.64	*p* = 0.02*	*r* ^2^ = 0.22 predicts 93%

*LR* likelihood ratio

*p < 0.05

## Discussion

The main finding in this cross-sectional study of BELFAST octo/nonagenarians, enlisted by Senieur protocol was that those who carried the mitochondrial J haplogroup had lower systolic blood pressure compared to non-J haplogroups, and this trend was also separately present in men and women who were octo/nonagenarians. Hypertension is the single most important risk factor for cardiovascular disease, stroke and Alzheimers disease, all of which contribute to early mortality, increased morbidity and poor quality ageing (Hajjar et al. 2007). There is a large amount of evidence to show that small decreases in blood pressure can achieve important effects for public health. A reduction in systolic blood pressure of 5 mmHg is estimated to reduce mortality to stroke by 14 %, mortality from heart disease by 9 % and all case mortality by 7 % (Lewington et al. 2002). Although mitochondrial haplogroups vary geographically and ethnically across the world, this finding in Caucasians is of particular interest in the light of recent findings of an association between the mitochondrial uncoupling protein 1 and blood pressure in Korean females which is independent of obesity and body mass index (Cha et al. 2008) and the identification of a mutation in mtDNA haplogroup G2a1 across three generations of a Chinese family which was associated with female hypertension (Luon et al. 2011).

There is increasing evidence that the various mitochondrial haplogroups have functional differences which seem likely to be related to reductions in ATP production, increased generation of reactive oxygen species and impaired calcium buffering (Postnov 2005), all of which may be related to central sympathetic central overstimulation (Postnov et al. 2007). Early studies linked various mtDNA haplogroups and mutations with clinical presentations associated with Leber’s optic atrophy, muscle storage disease and deafness, often with quite complex phenotypes (Wallace 2005). More recent publications have begun to show clearer links between mtDNA haplogroups and specific disease entities, i.e. osteoarthritis and the J mtDNA haplogroup, with J carriers having lower biomarkers of oxidative stress compared to non-J carriers (Fernandez-Moreno et al. 2010), and reduced osteoarthritic disease. There is also recent interest in the association of mtDNA haplogroups with age-related macular degeneration (Udar et al. 2009), where it appears that partial and thus subclinical deficiencies in mitochondrial energy production are associated with J, T and U mitochondrial haplogroups which are suggested to become more sensitive to age-related macular damage from a possible hexane-related metabolic pathway. A recent publication also suggests that the mtDNA J haplogroup had protective effects in modulating telomere length and nitric oxide production in carriers (Fernandez-Moreno et al. 2011). This finding resonated with earlier work showing that mtDNA haplogroups had altered oxygen-metabolising capacity in groups living at higher altitudes with mtDNA J haplogroup carriers having reduced DNA damage (Domínguez-Garrido et al. 2009). These studies provide increasing evidence supporting a mechanistic link between reduced oxidative stress status and carriers of the mtDNA J haplogroup. Similar findings are mirrored in the BELFAST study where glutathione peroxidase antioxidant enzymatic activity was lower in both serum and whole blood in octo/nonagenarians who carried the mtDNA J haplogroup and who also had co-existing lower blood pressure. The work of Rybka et al. 2011b shows similar changes with hypertension.

Higher glutathione peroxidase activity found in BELFAST octo/nonagenarians in association with higher blood pressure is in keeping with suggestions that oxidative stress plays an important patho-physiological role in the development of hypertension (Ceriello 2008; Lassegue and Griendling 2004; Redón et al. 2003). Oxidation by-products are considered to damage or inactivate endothelium-derived nitric oxide, an important vasodilator, and as a result cause reactive vasoconstriction. Although there is broad agreement of a relationship between oxidative stress and essential hypertension (Rodrigo et al. 2007), the interpretation of evidence is far from simple. Gpx is mostly reduced in situations where anti-oxidant stress is minimal, but increased where there is, for example hypertensive or cardiovascular stress linked with the inflammatory cascade (Harrison et al. 2003; Rea et al. 2011). Therefore a possible interpretation of the BELFAST data could be that J haplogroup survivors exemplify a low anti-oxidant stress model for Gpx and the GPx/GSH redox re-cycling reaction. Interestingly, reduction in GPx 4 activity in knockout mice, increased lifespan in the mice, with a mechanism relating to oxidative stress-induced apoptosis (Qitaoran et al. 2007), suggesting possible parallel mechanisms in humans.

The molecular pathway linking mitochondrial haplogroups and mutations with mechanisms and function in hypertension has become a bit clearer as a result of these publications and the recent work by Luon et al. 2011. Here, the tRNAmet4435A4G mutation in mitochondrial haplogroup G2a1 was responsible for maternally inherited hypertension across three generations of female family members in China. This mitochondrial mutation appears to reduce protein synthesis, with downstream impairment of the mitochondrial respiratory chain function, reduction of ATP production, increased reactive oxygen species production and enhanced hypertension susceptibility.

Albumin and bilirubin are the most abundant and important non enzymatic anti-oxidants in the body (Vítek and Ostrow 2009; Halliwell 1988) and their equilibrium might be expected to be disturbed in association with hypertension but we found no evidence to support this hypothesis in BELFAST octo/nonagenarians. We also found no changes for serum vitamin C, although folate sometimes considered a surrogate for Vitamin C and vegetable intake, was significantly higher in mtDNA J haplogroup carriers. Dietary vitamin C has been shown to increase antioxidant capacity (Cao et al. 1998), to associate with lower blood pressure (Myint et al. 2011; Ceriello 2008), but not to enhance longevity (Choi et al. 2004). Although Vit E was marginally increased in J haplotype carriers, we did not find that vitamins A and α and β carotenoids were different across mtDNA J haplogroup categories in BELFAST octo/nonagenarians unlike Semba et al. 2006 who demonstrated that low carotenoid micronutrients predicted frailty and mortality in community-living older women. However, our groups were small and follow-up to date has been limited.

Important limitations of this research are the cross-sectional nature of the BELFAST study and the numbers which may have reduced the power. However, 90 year olds are both the fastest growing sector of present day populations and the group about whom we know relatively little medically, socially and economically. It can be argued that they are of special interest because of their extreme age and because in this study they were well categorised by Senieur status (Rea et al. 2009; Rea 2010; Lighthart et al. 1984). Studies in this age group are extremely challenging because of perceived or real frailty and the understandable concern of the subjects themselves, relatives, researchers and Ethical Committees not to exploit, tire or over-ride the autonomy of the subject group (Samelson et al. 2008). For these reasons, some subjects contributed only anthropometric measurements or a limited amount of blood. Furthermore, catalase and superoxide dismutase have not been assessed as contributors to the enzymatic antioxidant profile in BELFAST octo/nonagenarians, and this leaves an important unknown aspect of the total anti-oxidant profile.

The findings in the BELFAST study are very preliminary but are potentially interesting. They will need to be replicated but do provide some support for the idea that carriage of the mtDNA J haplogroup may be associated with a lower blood pressure profile. The associated altered antioxidant profile, which has been identified could also play a role in a longevity phenotype (De Benedictis et al. 1999; Ross et al. 2001; Niemi et al. 2003), though as has been suggested by others, the J mitochondrial haplogroup may act out a different role depending on its associated nuclear and/or dietary or life-style microenvironments (Gomez and Hagen 2012). In terms of mechanisms Marcuello et al. 2009 recently described J haplogroup carriage in male Spanish subjects to be associated with a lower efficiency of the electron transport chain, diminished ATP and reactive oxygen species production suggesting possible decreased oxidative damage with this haplogroup. In an interesting follow-up study, this group noted that mtDNA haplogroup differences in oxygen energy production were negated by exercise, a clear example of a link between molecular pathways and environmental and life-style behaviours (Martínez-Redondo et al. 2010), while Rose et al. 2010 suggest a link between mitochondrial mutations and frailty markers. Rea et al. 2011 also suggest a link between hypertension, anti-oxidant stress and the inflammatory cascade related to infection. A further argument is that the enzymatic anti-oxidant system is much more likely to be under nuclear and/or mitochondrial genetic or metabolic fine-tuning and so more likely to be demonstrately changed in long-living species including man. We found no evidence that mitochondrial haplogroup T, which shares the same sub-root as J, was associated with lower blood pressure as found with the J mitochondrial group, directing attention to a possible role for T4216C, A11251G and C15452A mutation substitutions not shared with haplogroup T. Recent work also suggests that the J1 and J2 subtypes of mtDNA J haplogroup may have functional differences. These findings need to be replicated in ongoing studies of ageing such as the Genetics of Healthy Ageing study (Franceschi et al. 2007; Rose et al. 2010; Skytthe et al. 2011) where there is adequate power and sample availability to link mitochondrial haplotypes with lifestyle phenotypes.
